# Mini-review and situation report on the role and usefulness of nuclear medicine imaging for malaria

**DOI:** 10.1007/s00259-025-07443-4

**Published:** 2025-08-20

**Authors:** Janie Duvenhage, Jan Rijn Zeevaart, Mike Machaba Sathekge, Thomas Ebenhan

**Affiliations:** 1 Nuclear Medicine Research Infrastructure NPC, Pretoria, South Africa; 2 Radiochemistry, the South African Nuclear Energy Corporation (Necsa) SOC Ltd, Pelindaba, South Africa; 3 Department of Nuclear Medicine, University of Pretoria, Pretoria, South Africa

**Keywords:** *Plasmodium*, Severe malaria, Nuclear imaging, Positron-emission tomography, Cerebral malaria

## Abstract

Malaria remains one of the deadliest parasitic diseases globally. Delay or failure in treatment can lead to the development of severe malaria. Severe malaria, an understudied multisystem disease, affects the host’s organs and can lead to several syndromes and serious complications, some effecting life-long neurological and cognitive sequela. There is a lack in knowledge regarding the molecular mechanisms underlying severe malaria pathogenesis, and research has mostly relied on post-mortem studies and animal models, both of which lack translatability to human malaria. This review presents the clinical nuclear imaging techniques used in malaria. Although the presented radiopharmaceuticals have added value to understand some aspects of severe malaria, there has been in stagnation in development of more malaria-specific radiopharmaceuticals. This manuscript highlights the current limitations for implementing improved radiopharmaceuticals and provides valuable insights on how these challenges can be overcome.

**Supplementary Information:**

The online version contains supplementary material available at 10.1007/s00259-025-07443-4.

## Introduction

Despite remarkable progress in the global elimination, malaria remains as one of the most life-threatening parasitic diseases worldwide. Although malaria is entirely preventable and treatable, it remains responsible for half a million deaths annually [1]. Malaria is caused by protozoan parasites of the genus *Plasmodium* (*P*) of which five species are responsible for the disease in humans. *P. falciparum* is the most virulent species, responsible for causing the most lethal forms of human malaria [1]. Clinically, malaria can be categorised as uncomplicated or severe (complicated). Uncomplicated malaria (UM) represents the initial stage of the disease, typically presenting flu-like symptoms such as fever, chills, headache, and muscle aches. SM is a multi-system disease that affects various organs and can lead to several overlapping syndromes, including malaria-associated acute respiratory distress syndrome (MA-ARDS), cerebral malaria (CM), severe malaria anaemia (SMA), acute kidney injury (AKI), and splenomegaly [2]. Survivors of SM often suffer life-long neurologic or cognitive impairment which substantially decreases their quality of life, including speech difficulties, behavioural problems, trouble of movement, and loss of sight or hearing, and even dementia. Both parasite and host factors contribute to the development of SM [2]. Therefore, understanding host-parasite interactions is critical in designing targeted interventions and predicting severe malaria syndromes. Gaining insight on these interactions has traditionally relied on the use of animal models, biochemical biomarkers and post-mortem investigations. Each of these conventional investigations, however, has limitations. Although animal models have provided vital information regarding SM, the translatability of these findings to human infections is limited. Biomarkers are only able to diagnose single syndromes, and post-mortem analyses lack the ability to provide information regarding events that occurred before death. In the clinical setting, in vivo imaging has allowed the repeated capturing of infectious diseases in real time and has helped to obtain unique insights into numerous disorders and pathologies. These established non-invasive imaging techniques can be translated to malaria, permitting the visualisation of processes during malaria infection and observation of the host-parasite interactions that govern pathogenicity. Ultimately, the use of these techniques would assist in gaining a more thorough understanding of SM.

Observations indicate that the research area for developing malaria-related radiopharmaceuticals for positron emission tomography (PET), or single-photon emission computed tomography (SPECT) appears to be stagnant. Historically, radiopharmaceuticals have demonstrated the value of clinical imaging techniques in the context of symptomatic malaria. However, nuclear medicine techniques that enable the study of functional or metabolic aspects of malaria have been the particular focus. While magnetic resonance imaging (MRI) and ultrasonography (US) have been commonly used in malaria research, there is a need to develop improved, disease-specific imaging strategies for malaria. This manuscript aims to address the challenges associated with these developments and explore potential new avenues for nuclear medicine techniques.

## The life cycle of *Plasmodium falciparum*

**Fig. 1:**
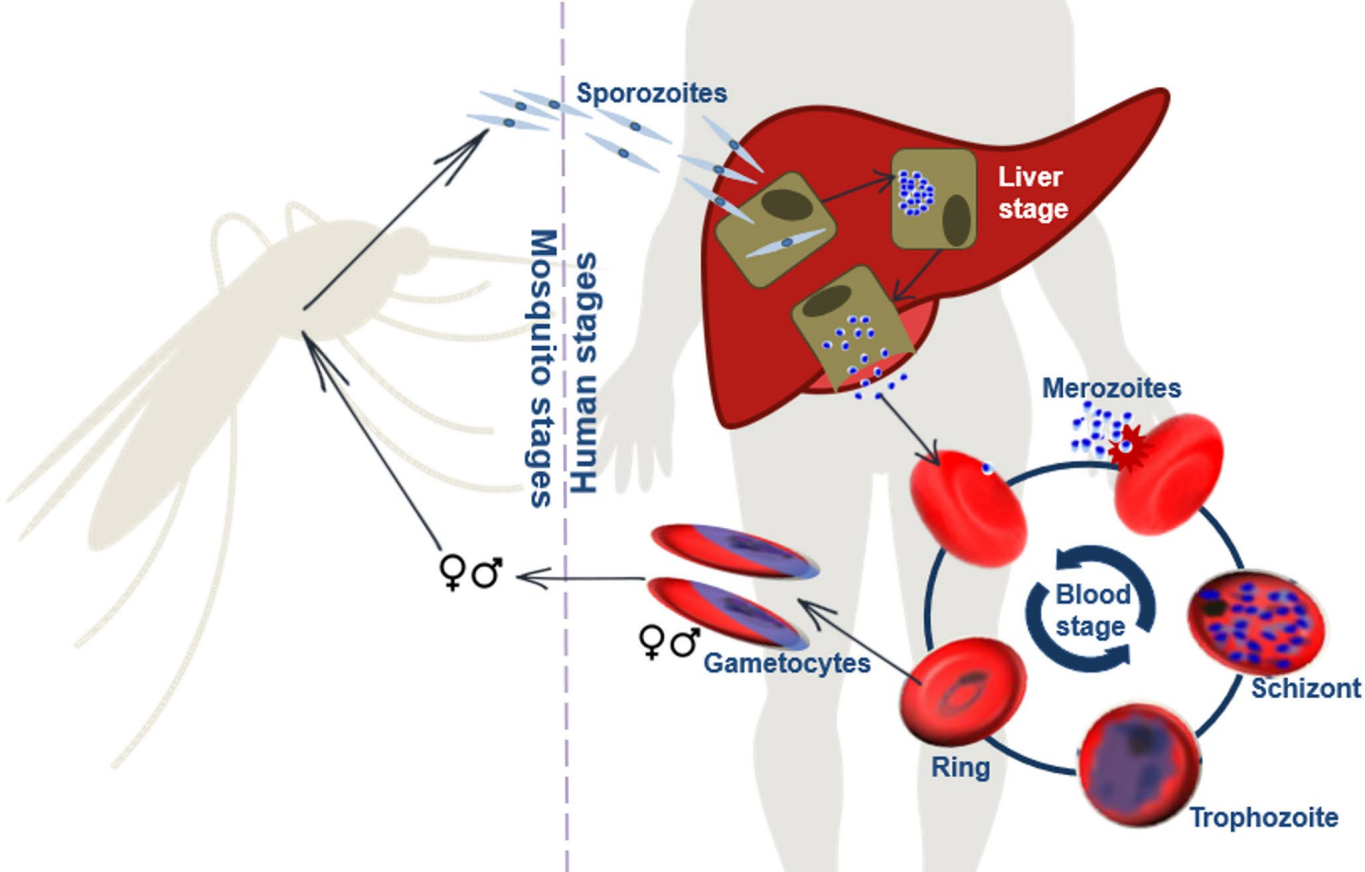
The life cycle of ***Plasmodium falciparum***. The parasite used both a human host and mosquito vector to complete its life cycle

The life cycle begins with the bite of an infectious Anopheles mosquito where sporozoites enter the human host’ blood stream and travel to the liver to invade liver cells and mature into merozoites (Fig. 1) [3]. The liver cell bursts and releases the merozoites into the blood stream to which then infect erythrocytes, initiating the asexual intra-erythrocytic developmental stage. After entering the erythrocyte, the parasite develops from a ring stage into metabolically active trophozoites and thereafter undergo nuclear divisions to produce multinucleated schizonts consisting of multiple merozoites. These merozoites get released into the blood stream when the erythrocyte bursts and then reinvade new erythrocytes and thereby continuing the asexual replication cycle.

A small portion of the asexual parasites undergo gametocytogenesis where they enter the sexual reproduction cycle forming male and female gametocytes that matures while sequestering in the bone marrow. Once mature, these gametocytes enter the host’s blood stream where they can again be taken up by a feeding mosquito. Mature male and female stage gametocytes develop into a macrogamete and microgamete respectively that fuse with each other to form a zygote. The zygote transforms into an ookinete which travels to the midgut of the mosquito and matures into oocyst. The oocyst produces sporozoites which can then be injected into the human host when the mosquito feeds, starting the cycle again.

## Clinical imaging techniques support malaria diagnostic and disease symptomatic

Historically, multiple anatomical imaging techniques have been utilised to diagnose and better understand malaria including radiography (X-ray), US, computed tomography (CT), and MRI. Of these techniques, US and MRI have made valuable contributions to malaria diagnosis and patient monitoring.

Chest X-rays are usually efficient to identify MA-ARDS, ALI, pulmonary oedema, and also provided proof that *Plasmodium* alone is capable of causing pneumonia [4–6]. Radiological signs of Plasmodium-associated pneumonia include increase in interstitial markings, identifying bilateral diffuse infiltrates, increased broncho-vascular markings, hyperinflated lungs and effusion, perihilar patchy consolidation, and other markings similar to ARDS [7].

Point-of-care lung ultrasound (US) is a direct, sensitive, and non-invasive technique that was often used to support the diagnosis of pulmonary complications in individuals with malaria including MA-ARDS [8]. Abdominal pain in patients with SM is common and is largely due to malarial gastroenteritis, drug-induced gastritis, or hepatitis [9]. Abdominal US enabled the identification of the gallbladder wall thickening to the development of acute acalculous cholecystitis (AAC) [9]. Hand-held US devices have proven an effective and accessible method to determine spleen- and liver size as part of the diagnostic algorithm for SM [10].

Abdominal CT has been used to study other malaria syndromes and complications, but US devices provide comparable information for malaria-induced abdominal complications and thereby avoid the need for more costly CT imaging [10–13]. As CT scans underestimate disease burden compared with clinical findings and post-mortem examinations, using CT alone is not suitable to study malaria pathophysiology [14].

MRI has contributed substantially to cerebral malaria diagnosis; its longstanding literature track-record compromising of numerous significant reports regarding CM (Summary: Table 1), including, but not limited to cerebral swelling; brain stem herniation and demyelination; ischaemic lesion detection in the central splenium of the corpus callosum; focal cortical and subcortical infarcts in the cerebellum and cerebrum, and abnormalities in basal ganglia and cerebral cortex [15]. Overall, MRI has revealed distinct differences in pathogenic patters in paediatric and adult manifestations of CM [16]. For example, increased brain volume led to augmented cerebral pressure and brain stem herniation in fatal paediatric CM, where severe hypoxia was associated with fatal outcome of adult CM. MRI studies in the non-fatal cases of paediatric CM suggest that vascular congestion, autoregulatory dysfunction and microhaemorrhages contributes to brain swelling pathogenesis. MRI was useful to point out that brain swelling and vasogenic oedema, a result of breakdown of the blood brain barrier, are more common and likely to cause fatalities among children.

**Table 1:** Connecting clinical presentation of cerebral malaria with MRI imaging

**Symptoms/Clinical manifestations**	**MRI finding**	**Affected brain regions**	**Conclusions of findings**	**MRI sequences**	**Ref**
**Adult Cerebral Malaria**					
Reduced consciousness	Oedema (cortical, vasogenic, cytotoxic) Venous congestion	Swelling (mostly posterior) BBB	Swelling due to impairment of BBB Venous congestion causing sequestration of iRBCs	T2 FLAIR	[17]
Organ dysfunctions (hepatomegaly, jaundice, splenomegaly) Neurological sequalae (coma, focal seizures, convulsion) Arrythmia/Anaemia Breathing issues	Oedema progressed from acute CM Vascular occlusion Herniation (Foramen magnum)	Brain stem Intracerebral blood volume	Swelling probably due to sequestered iRBCs and vasodilation rather than from oedema	T1W T2W	[18]
Fevers Neurological sequelae (coma, memory loss, seizures)	Central pontine myelinolysis (CPM)	Brain stem	CPM suggested (caused by ischaemia or the toxic effect of the parasitised red blood cells in the cerebral microvasculature leading to capillary occlusion and damage)	T1W T2W FLAIR	[19]
Death Neurological sequelae (coma, convulsions)	Mild cerebral swelling (without vasogenic oedema) Mildly increased lactate levels	Basal ganglia Posterior fossa	Brain swelling insufficient to cause coma or death (also not due to vasogenic- or cytotoxic oedema) No differences between fatal and non-fatal infection	T1W T2W FLAIR GRE DWI MRS _GM_	[20]
Flu-like symptoms (fever, chills, vomiting) Neurological sequelae (seizures into epilepticus, loss of power in upper limb)	Haemorrhagic infarct in parietal region Oedema in WM Hyperintensities (parietal and insular cortex)	Posterior lobe (frontal, parietal) Subcortical WM Thalamus Insula	Nd.	T1W T2W FLAIR	[21]
**Paediatric Cerebral Malaria**					
Renal dysfunction Neurological sequelae (seizures, coma)	Moderate oedema Abnormal basal ganglia (globus pallidus, putamen) Abnormalities in pons and brainstem Haemorrhages	Cortex Basal ganglia Thalamus Brainstem (pons) Corpus callosum Cerebellum	Haemorrhages caused by parasite sequestration Brain swelling in non-fatal cases possibly caused by vascular congestion, auto-regulatory dysfunction and micro-haemorrhages	DWI SWI T2 Gd	[22]
Decreased consciousness Death	Increased brain volume Absence of cytotoxic oedema Decreased prepontine CSF levels (fatal cases)	Prepontine Posterior cerebral predominance Thalamus	Increased brain volume uncommon in non-fatal CM (increased intracranial pressure may contribute to the CM pathogenesis in children)	T1W T2W	[23]
Comatose Death	Isolated subcortical white matter diffusion restriction (IWMDR) (only in subcortical regions)	Subcortical WM Corpus callosum Deep GM Cortical GM	IWMDR restriction associated with less severe disease and better outcomes	DWI	[24]
**#**) Neurological sequela (behavioural disorders, development delay, epilepsy, seizures)	Cerebral atrophy Focal lesions	WM (Subcortical, peri-ventricular) Thalamus Basal ganglia Cortex	Nd.	T2W DWI	[25]

*CM* Cerebral malaria; *BBB* blood brain barrier; *P. falciparum* *Plasmodium falciparum*; *iRBC* infected- red blood cell; *T1W* T1 weighted; *T2W* T2 weighted; *FLAIR* fluid-attenuated inversion recovery; *DWI* diffusion weighted imaging; *MRS* magnetic resonance spectroscopy; *GRE* gradient Echo; *Gd* Gadolinium-contrasted; *SWI* susceptibility-weighted imaging; *GM* Grey matter; *WM* white matter; *#* Followup MRIs of paediatric CM survivors with neurological sequela

Brain MRI in adults with SM identified mild brain swelling, that would be insufficient to cause coma or neurological symptoms. Cytotoxic oedema, possibly related to ischaemia, or acute demyelination are associated with death in adults.

MRI has been used to expose cerebral abnormalities in individuals with UM and no signs of standard neurological abnormality, highlighting that UM may have a currently unrecognised impact on neurological diseases and disabilities. Table 1 highlights studies where MRI has been useful to study the clinical presentation of CM. It is impactable that in patients with acute, but uncomplicated malaria (e.g., no evident neurological disorder, vital organ dysfunction, or other severe manifestations of infection) MRI can identify fatal lesions in the splenium of corpus callosum – crucial findings that emphasise that uncomplicated malaria may already have affected the onset of neurological diseases and other disabilities [26].

## Nuclear imaging provides prospects for research to explore malaria on a molecular level

Nuclear imaging utilises radiopharmaceuticals to visualise diseases such as malaria and other infection. Modern scanners in Nuclear Medicine Departments are combining a CT or even MRI with either SPECT or PET to yield hybrid images.

**Fig. 2:**
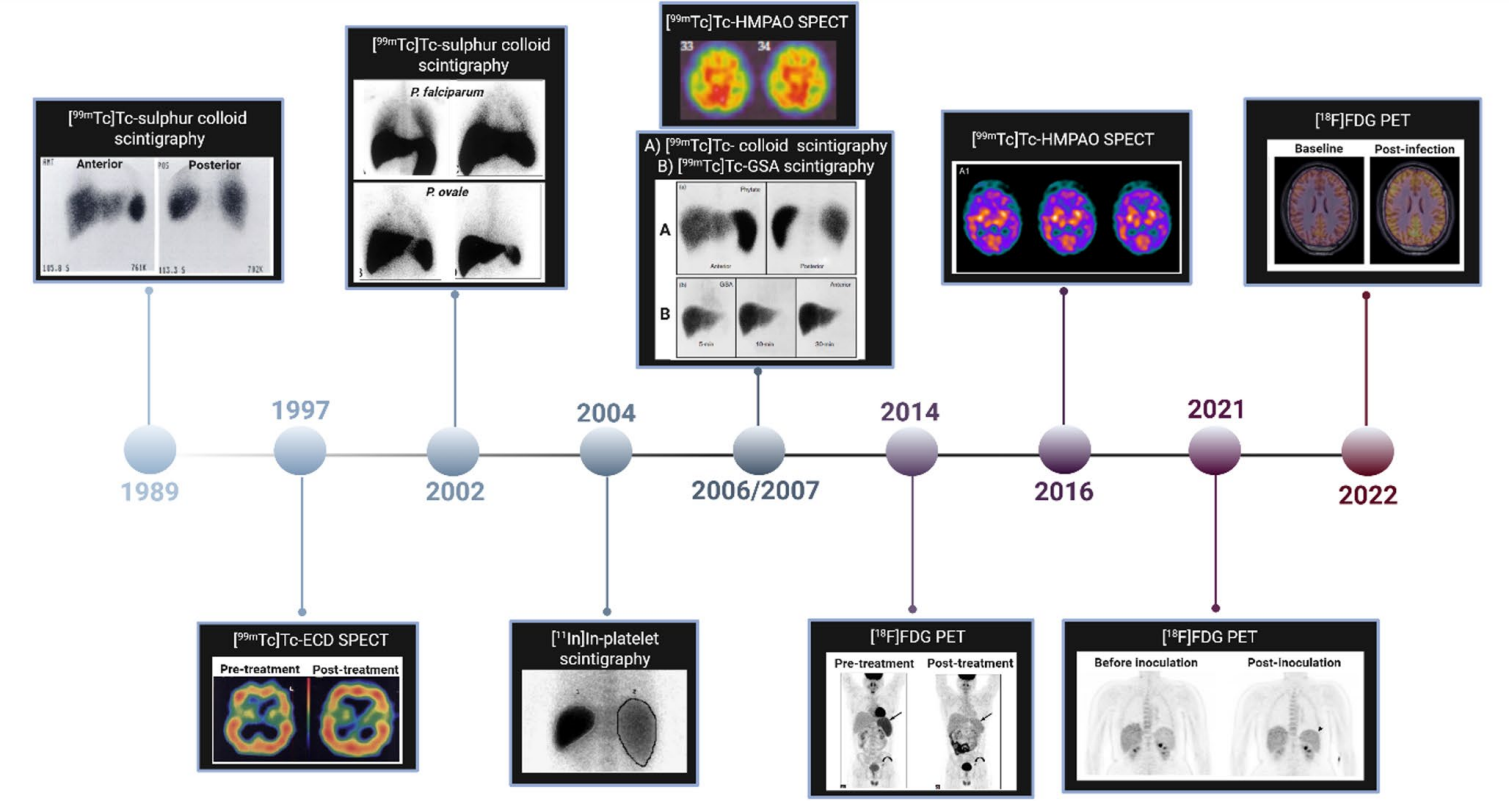
Chronology of nuclear imaging techniques used for in vivo clinical investigations of malaria. [^99m^Tc]Tc-ethyl cysteinate dimer ([^99m^Tc]Tc-ECD); [^99m^Tc]Tc-Galactosylated serum albumin ([^99m^Tc]Tc-GSA); [^99m^Tc]Tc-hexamethyl-propylene-amine oxime ([^99m^Tc]Tc-HMPAO); 2-deoxy-2-[^18^F]fluoro-D-glucose ([^18^F]FDG) [28–37]. Figure created by licences software (BioRender.com)

In the clinical setting the use of Nuclear Medicine for imaging, the diagnosis of malaria has been limited to a few instances due to the complexity of the disease and the urgency for treatment once a patient is admitted (Fig. 2).

[^99m^Tc]Tc-sulphur colloids ([^99m^Tc]Tc- colloids), a radiopharmaceutical formulation that is actively phagocytosed by hepatic Kupffer cells and splenic macrophages, can been utilised to track reticuloendothelial system (RES) activity. In 1976, [^99m^Tc]Tc-colloids scintigraphy was performed on a patient with suspected hepatitis, who was subsequently diagnosed with *P. falciparum* malaria [27]. The scintillation scan showed uneven uptake of [^99m^Tc]Tc-sulphur colloid by an enlarged liver, a severely enlarged spleen, and significant uptake by the lung as a result of increased RES activity. Despite being an accidental finding [^99m^Tc]Tc-sulphur colloid imaging was suggested for detecting malaria-related organ pathology as *Plasmodium* infection stimulates RES cells.

The *first confirmed case study of malaria* using nuclear imaging was reported in 1989 where [^99m^Tc]Tc-sulphar colloids uptake was determined in a patient diagnosed with *P. vivax* malaria infection [28]. This study revealed increased splenic uptake, moderate colloid uptake in the lung but negligible bone uptake. A follow-up scan after treatment indicated that all abnormal organ intensities were resolved. [^99m^Tc]Tc-colloid imaging was hereby employed to indicate changes in liver size and Kupffer cell distribution but this technique lacks to reflect any hepatocyte functionality. Thus, SPECT imaging using [^99m^Tc]Tc-galactosylated-serum-albumin ([^99m^Tc]Tc-GSA) as a receptor-mediated technique that provides information on hepatic parenchymal function was considered. In 2006 Lee *at al.* compared [^99m^Tc]Tc-GSA and [^99m^Tc]Tc-colloid liver scintigraphy to understand the pathophysiology of liver involvement and to evaluate differential engagement of liver cells in the hepatic pathophysiology of the malaria life cycle [33]. The [^99m^Tc]Tc-GSA images were mostly normal, while the [^99m^Tc]Tc-phytate-colloid scintigraphy showed moderate to severe alteration of RES function. The study therefore demonstrated that [^99m^Tc]Tc-GSA and [^99m^Tc]Tc-colloid scintigraphy were complimentary and that [^99m^Tc]Tc-GSA scintigraphy can be used to differentiate between RES involvement and direct hepatocellular tissue damage caused by malaria infection.

Occasionally, malaria patients may also suffer from pulmonary obstruction where Nuclear Medicine techniques are advised [38, 39]. Ansty et al. (2002) used [^99m^Tc]Tc-sulphur colloid to assess pulmonary phagocytic cell activity in *P. falciparum*,* P. ovale*,* and P. vivax* -infected individuals by means of lung scintigraphy; an increase in pulmonary phagocytotic activity was identified in *P. falciparum* malaria, while only moderate signal increases were found in *P. vivax* and *P. ovale* malarias [30]. Additionally, alveolar epithelial permeability was assessed by measuring pulmonary clearance of aerosolised ^99m^Tc-labelled diethylenetriaminepentacetate ([^99m^Tc]Tc-DTPA); however, there was no significant deviation between healthy and malaria-infected individuals. The authors concluded that the causes for the differential pulmonary physiology between the latter three malaria parasite species were: (i) airflow obstruction; (ii) impaired ventilation; (iii) reduced gas transfer; and (iv) and increased pulmonary phagocytotic activity.

[^99m^Tc]Tc-bicisate, [^99m^Tc]Tc-ethyl cysteinate dimer ([^99m^Tc]Tc-ECD) and [^99m^Tc]Tc-hexamethyl-propylene-amine oxime ([^99m^Tc]Tc-HMPAO) are radiopharmaceuticals that are capable of studying brain perfusion with SPECT [40]. By imaging the regional cerebral blood flow, these tracers have proven useful for the diagnosis of psychiatric illnesses, Alzheimer’s disease, dementias, and traumatic injuries [40]. In 1997, a patient with CM underwent SPECT imaging after administration of [^99m^Tc]Tc-ECD to assess differences in cerebral perfusion [29]. The scan demonstrated a [^99m^Tc]Tc-ECD uptake pattern that was consistent with focal hypoperfusion in the symptomatic hemisphere. Upon discharge, the patient showed no signs of neurological sequelae in line with normal cerebral haemodynamic revealed by the [^99m^Tc]Tc-ECD-SPECT scan. Transcranial cerebral oximetry was utilised to approve the accuracy and value of [^99m^Tc]Tc-ECD-SPECT towards its disease-monitoring capabilities and, thereby, demonstrate the utility of this method as a non-invasive tool for measuring malaria-related vascular dysfunction and perfusion. Two studies have reported the evaluation of brain perfusion in CM in severely-ill patients by [^99m^Tc]Tc-HMPAO SPECT [32, 35]. In both cases, the patients suffered from post-malaria neurological syndromes. Moreno-Caballero et al.. (2016) reported heterogeneous and diffuse cortical hypoperfusion without clear regional tomographic predominance and discreet striatal asymmetry [35]. This pathological tracer distribution combined with the absence of structural changes (observed with MRI) support that a disruption in the cerebral microvasculature, possibly due to parasite sequestration, may be the main trigger of neurological symptoms. Brian perfusion SPECT were able to verify the reperfusion in all affected areas in the brain after treatment. Likewise, in the study by Hsieh et al. (2006), no structural alterations were observed through MRI, with [^99m^Tc]Tc-HMPAO SPECT indicating hypoperfusion in both cerebral hemispheres and especially in the bilateral temporal and parietal lobes [32]. The authors suggested possible causes including immune factors; cerebral sequestration of parasite-infected erythrocytes; encephalitis due to viral coinfection; or possible side effects of antimalarial medication. Although studies reporting brain perfusion SPECT for individuals with CM are limited, it does prove to be a useful tool to study CM pathophysiology providing the ability to detect alterations in brain perfusion, even in the absence of structural changes. Nuclear imaging strategies involving radiolabelled blood elements are readily available and routinely utilised. As malaria is a blood-borne disease, tracking associated haematological abnormalities is considered a valuable approach to study the disease. Thrombocytopenia is one of the many hematologic abnormalities associated with malaria. Scintigraphy has previously been used to explore the kinetics and sites of thrombocyte sequestration in patients with early-stage malaria and thrombocytopenia by means of tracking [^111^In]In-labelled platelets noninvasively using scintigraphy imaging [31]. In this study, local sequestration was not identified as the main cause of thrombocytopenia. Several mechanisms have been proposed for the low platelet counts associated with malaria, however, studies have excluded parasite sequestration as the potential mechanism by demonstrating that due to platelet-associated parasite killing, platelet-erythrocyte complexes form a major part of the total platelet count in malaria-infected individuals and may thereby contribute to thrombocytopenia [41].

Imaging using 2-deoxy-2-[^18^F]fluoro-D-glucose-PET ([^18^F]FDG-PET) has been useful for diagnosing and monitoring treatment efficacy in various inflammatory and infectious diseases [42]. In 2014, malaria was incidentally detected by [^18^F]FDG-PET/CT in a patient that was routinely scanned for relapse of Hodgkin’s lymphoma which is now hallmarked as the *first [*^*18*^*F]FDG-PET/CT case study* clearly confirmed by a blood smear for *P. vivax* infection [34]. A follow-up scan of the patient after antimalarial treatment revealed normalised spleen size and [^18^F]FDG uptake. Recently, [^18^F]FDG-PET/MRI was used in a small clinical trial investigation to identify early-on differences between *P. vivax* and *P. falciparum* infections [37, 43]. In this studies, healthy malaria-naïve participants underwent baseline [^18^F]FDG-PET/MRI seven days before they were inoculated with approximately 600 viable *P. vivax* or approximately 2800 *P. falciparum*-infected erythrocytes. Quantitative polymerase chain reaction (qPCR) assays were used to monitor peripheral blood parasitaemia and the second [^18^F]FDG-PET/MRI scan was performed near the peak of parasitaemia within 24 h before antimalarial treatment. An elevated splenic [^18^F]FDG signal was observed in participants with induced *P. vivax* and *P. falciparum* infections, with a more prominent [^18^F]FDG uptake seen for *P. vivax* infections [36]. Signal absence was observed in the bone marrow and in the liver for both *P. vivax* and *P. falciparum* infections, possibly indicating tissues do not harbour a large parasite biomass or do not provoke a prominent metabolic response in the initial infection stage [36]. Brain PET/MRI demonstrated higher inter-scan variability for [^18^F]FDG uptake in individuals with *P. falciparum* infection and limited variability in the case of *P. vivax;* however there was no significant difference in base-line and post-inoculation tracer uptake [37]. These results could propose that differences between *P. falciparum* and *P. vivax* tropism in the brain may arise very early in infection. Although these results may suggest that glucose metabolisms might be more affected by *P. falciparum* infection compared with *P. vivax* infection, the small population size and non-specificity of [^18^F]FDG limits the validity of this conclusion.

### Assessment of current clinical tools for nuclear imaging of malaria

**Fig. 3:**
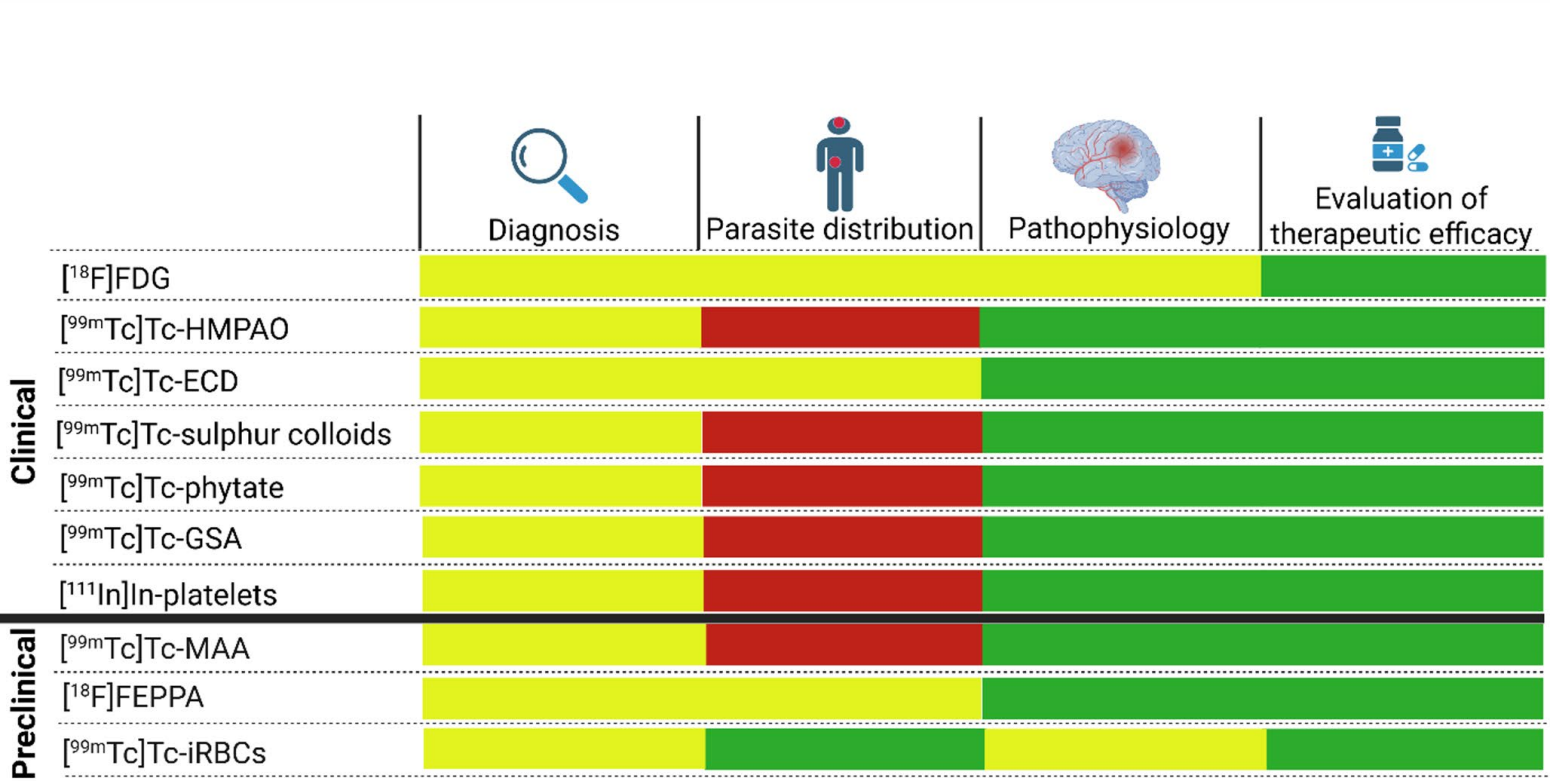
The value of current (are recently proposed) nuclear imaging techniques used in malaria. In this diagram, the value of the individual tracers is assessed according to their current or potential usage in malaria diagnosis, determining parasite distribution, studying pathophysiology of the disease, and evaluating therapeutic efficacy of anti-malarial compounds. Tracers used clinically include 2-deoxy-2-[^18^F]fluoro-D-glucose ([^18^F]FDG), [^99m^Tc]Tc-hexamethyl-propylene-amine oxime ([^99m^Tc]Tc-HMPAO), [^99m^Tc]Tc-ethyl cysteinate dimer ([^99m^Tc]Tc-ECD), [^99m^Tc]Tc-sulphar colloids, [^99m^Tc]Tc-phytate, [^99m^Tc]Tc-Galactosylated-serum-albumin ([^99m^Tc]Tc-GSA), and [^111^In]In-platelets. Tracers evaluated preclinically are [^99m^Tc]Tc-macroaggregated albumin ([^99m^Tc]Tc-MAA), [^18^F]fluoro-N-(2-(2-fluoroethoxy)benzyl)-N-(4-phenoxypyridin-3-yl) acetamide ([^18^F]FEPPA) and [^99m^Tc]Tc-labelled infected- red blood cells ([^99m^Tc]Tc-iRBCs)

we analysed the currently available tracers concerning their ca uracy to reflect the disease burden (parasitic distribution), reflection of organ pathology and/or treatment efficacy, each one still has their own advantages and potential in improving knowledge about the malaria (Fig. 3).

## Current situation concerning the usefulness of nuclear imaging techniques for malaria

Malaria remains a significant global health challenge, and this situation report aims to assess whether the scientific community is advancing toward integrating newer technologies, such as nuclear imaging, to develop innovative insights and solutions for malaria treatment and prevention.

**Fig. 4:**
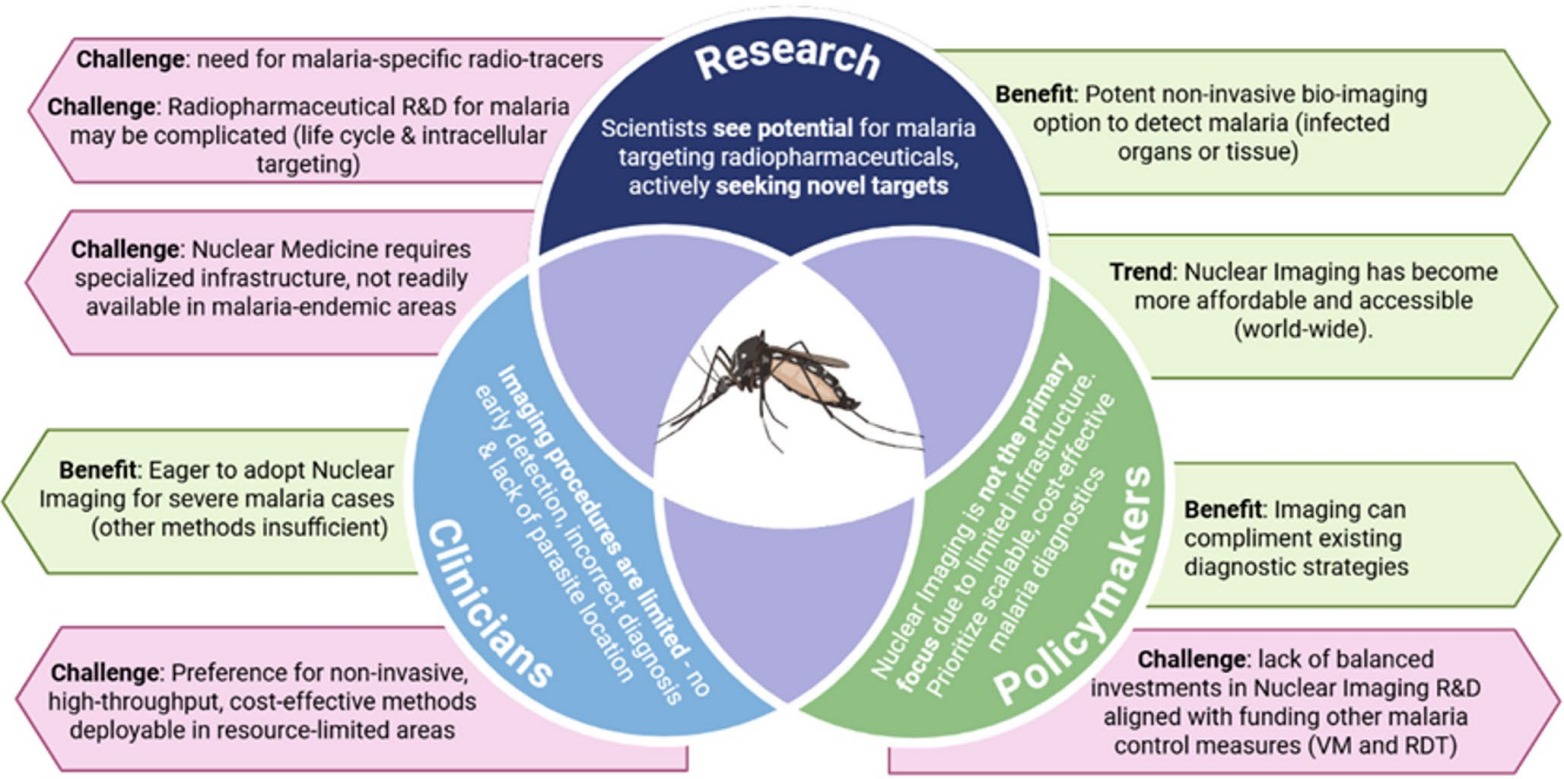
Stakeholder perspectives on nuclear imaging of malaria [45]. Research and development (R&D); rapid diagnostic testing (RDT); vector management (VM)

To establish a comprehensive evaluation, this report draws on reviewed literature on malaria, alongside data from global health organisations, regulatory bodies, product developers, and research institutes. These sources help defining the broader challenges associated with malaria, supported a comparative assessment of nuclear medicine techniques *versus* other imaging modalities used in malaria diagnostics (Table 2) and by a stakeholder perspective (Fig. 4).

**Table 2:** Comparative summary of nuclear medicine techniques with other imaging modalities used in malaria diagnostics [44]

**Modality**	**Sensitivity**	**Specificity**	**Cost**	**Infrastructure requirements**	**Suitability for low-resource settings**
NI (PET/SPECT)	Moderate	High	Very High	Advanced NI centres (central or mobile units)	Low
Microscopy	Moderate	High	Low	Basic lab setup needed	High
PCR	High	High	Moderate	Molecular lab	Moderate
RDT	Moderate	High	Moderate	Point-Of-Care Canter	High
AI-assisted imaging	High	High	Moderate	Requires digital infrastructure	Moderate

*NI* Nuclear imaging; *AI *artificial intelligence; *RDT* rapid diagnostic testing; *PCR* polymerase-chain reaction methods; *PET* positron emission tomography; *SPECT* single-photon emission tomography

Being **overall problematic**, malaria is a typical disease where diagnosis is critical in early clinical settings to ensure timely and appropriate treatment, reduce complications, and prevent the spread of the disease. As malaria symptoms — like fever, chills, and headache — can mimic other diseases, accurate diagnostic procedures (and parasite-specific tests) have to identify whether malaria is actually present. By accurately confirming cases before treatment, diagnostics can prevent the unnecessary use of antimalarial drugs, reducing the risk of parasite resistance. We know that for severe cases, techniques that allow regular patient monitoring during treatment can help to prevent complications like organ failure or CM. In malaria endemic regions where high disease burden calls for urgent, accurate diagnosis can also help to curb outbreaks and improve control measures. Based on a recent systematic analysis there is adequate evidence to suggest that malaria detection or diagnosis will progress significantly in the next decade [44]. However, despite its growing clinical relevance but contrary to other disease areas of Nuclear Medicine, the current role and effectiveness of Nuclear Medicine in malaria imaging are not clearly defined to date.

Given the **clinical need**, an innovative technology such as Nuclear Imaging can offer unique insights into malaria pathophysiology. However, its practical application supporting routine diagnostics remains limited due to high costs, infrastructure challenges, and the lack of specific radiotracers. Currently, microscopy of blood smears remains the most widely used diagnostic method, while polymerase-chain reaction-based methods, rapid diagnostic testing, and artificial intelligence-assisted imaging are emerging as powerful alternatives with improved sensitivity and automation potential.

Stakeholder consensus suggests that advances in our understanding of malaria-related organ pathologies are improving medication strategies, enabling effective disease management. The benefit of nuclear imaging could justify the required efforts; however, the effectiveness of nuclear imaging techniques is constrained by current radiotracers, limiting their diagnostic accuracy. Essentially, we can only diagnose and treat what we are able to visualise correctly. Thus, advancements in drug- and tracer development are essential to expanding malaria’s diagnostic toolbox and facilitating antimalarial drug discovery.

### The central issue – how to improve specialised imaging concerning malaria diagnostics

Despite ongoing challenges, specialised imaging research for malaria diagnostics remains a crucial area of exploration, with promising strategies needed to overcome technical limitations and enhance disease detection capabilities. Despite the historical contribution of radiopharmaceuticals in clinical imaging of malaria symptomatically, there is undeniable stagnation in the development of novel, more malaria-specific radiopharmaceuticals for PET- or SPECT imaging. This stagnation limits the potential for advanced nuclear medicine techniques to study the functional and metabolic aspects of malaria, which can be crucial for improving diagnosis or monitoring treatment efficacy. We can already agree that bridging this gap requires advancements in tracer development, accessibility, cost reduction, and interdisciplinary collaboration. While routine malaria diagnostics may not yet benefit from nuclear imaging, targeted research and infrastructure expansion can unlock its potential for specialised applications.

For now, by purely looking at the published evidence, we have to acknowledge that nuclear medicine imaging studies on malaria were quite limited, and, among the reported studies, many are not from peer-reviewed journals, sometimes very backdated and/or contain only preliminary findings. None of the nuclear medicine imaging tracers presented in the manuscript are evidently disease-specific (i.e., allow for malaria parasite imaging) but mostly target host factors responding to malaria infections, and in turn diagnose single syndromes or events, which are also present in other infections. In areas where malaria infections are endemic, infections with other pathogens can randomly occur before or whilst presenting for malaria imaging. The effect of diagnosing opportunistic infections in patients with malaria can be too complex; therefore, in these cases, proper initial blood culture testing should be performed prior to a referral for imaging. We can agree that, in malaria-confirmed patients, routine [^18^F]FDG-PET(MRI or CT) has some value to identify malaria infections at an early stage, but more validation is desired to confirm its effectiveness for early detection. Non-invasive PET- and SPECT imaging may further help visualising infected organs, such as the spleen and liver, where malaria parasites may accumulate; however, the precise localisation of active parasite clusters has not been achieved to date. Consequently, Nuclear Medicine’s advanced practises such as differentiating malaria from other conditions, tracking disease progression, and innovative image-guided backing for treatment strategies will remain crucial shortfalls.

### Evaluating current limitations and potential solutions in nuclear medicine-based malaria diagnostics

As outlined in the mini-review section, while nuclear imaging holds promise for malaria diagnostics, its extended consideration is hinged on addressing critical objectives (non- exhaustive list) such as:

- **To improve radiopharmaceutical development to given clinical needs** – novel, more disease-tailored malaria radiopharmaceuticals must allow for improved imaging accuracy and sensitivity. Current challenges using available radiopharmaceuticals include initial disease diagnosis and visualisation of parasitic distribution. Identifying new cellular targets could be crucial for developing more effective imaging strategies. Prioritising research into selective imaging of malaria-unique pathways may lead to significant improvements.
- **To master imaging tissues of low parasite density** – the reduced concentration of parasites in early infection stages complicates accurate detection through nuclear imaging. Future imaging strategies must ensure sufficient infectious tissue contrast, target-bioavailability, and efficient clearance from non-infected tissues. Tissue preloading (pre-targeting) strategies may be advisable.
- **To address the inadequate integration of nuclear imaging for severe malaria cases** – common diagnostic approaches often exclude nuclear imaging in advanced manifestations like CM. The disease’s intricate pathophysiology may surpass the limitations of existing non-specific nuclear imaging techniques, leading to considerable overlap with other neurological conditions. Efforts must lead to enhancing imaging specificity and refined detection methods to warrant clinical utility.
- **To enhance access to advanced technology and expertise** – malaria-endemic regions often face challenges due to insufficient infrastructure and a shortage of trained personnel to support nuclear medicine applications. However, the rapid technological advancements in this field are expected to expand the global footprint of centralised diagnostic hubs and mobile scanner units. The proven success of nuclear imaging in diagnosing other diseases is likely to drive its adoption for imaging complex infectious diseases like malaria. Health organisations and nuclear imaging associations play a crucial role in bridging these gaps by facilitating specialised training programs and streamlining the distribution of radioisotopes and ready-to-inject radiopharmaceuticals.
- **To tackle the burden of high costs** – The financial and logistical challenges tied to PET/SPECT technology hinder the feasibility of playing a bigger role in routine malaria screening. Collaborations between governments, health organisations, and private companies can drive funding and resource allocation for nuclear imaging in malaria diagnostics. However, a multi-pronged approach is advisable including investments in more affordable imaging techniques (including malaria-specific radiopharmaceuticals), strategic infrastructure expansion in malaria-endemic regions, technologic optimisation yielding in lower-cost options for imaging systems, and realistic streamlining of capacity building, training and supply (e.g., service) chains.

Despite the interconnected nature of these objectives, we will focus on providing more information and insights to overcome the deficiencies in tracer development and the complexities surrounding the integration of nuclear imaging for severe malaria cases – areas where tangible advancements in diagnostic capabilities are currently achievable.

To identify **potential solutions in nuclear imaging-based malaria diagnostics** this report will elaborate on various relevant topics which concern: a) using the parasite life cycle as a guide to list new targets and potential radiopharmaceuticals, B) outline good practices in using malaria animal (imaging) models in malaria research, and C) summarise clinical trial challenges attributed to imaging of malaria.

#### New targets and potential radiopharmaceuticals

Multiple stages of the *Plasmodium’s* life cycle are occurring in the human host, however, malaria presents several unique molecular targets in humans that could be leveraged for the development of new radiopharmaceuticals. These targets are primarily associated with (1) the *Plasmodium* parasite’s life cycle (see previous section) itself, (2) parasite interaction with its primary host cell (iRBCs), and (3) the malaria-triggered, complex host immune response.

Designing radiopharmaceuticals around malaria-infected red blood cell (iRBC) targets requires innovative strategies in molecular imaging. It is already well documented that iRBCs undergo significant molecular changes, offering potential targets for radiopharmaceutical development. These molecular targets could pave the way for improved malaria imaging using nuclear medicine techniques.

Despite the extensive list of potential targets, the majority have not yet been explored for in vivo imaging of malaria pathology. However, it is plausible that existing radiolabelling strategies and radiosynthesis protocols originally developed for other diseases could be adapted for malaria-specific compounds (for example mAbs, peptides, small molecules, or drugs). Many of these biomolecules, already utilised in radiopharmaceutical research and development, may offer viable pathways for malaria imaging. The development of malaria-specific imaging agents demands a rigorous approach, integrating comprehensive evidence from non-radioactive studies on their mechanisms of action. Ensuring optimal specificity and efficacy in detecting infected red blood cells must be closely correlated with their associated pathological changes, particularly in cases of sequestration within deep tissues. The extended field of radiopharmaceutical development for imaging of infectious diseases can also provide meaningful insights, which the following section will elaborate on. Furthermore, advancements in radiopharmaceutical development for imaging infectious diseases provide valuable insights that can be leveraged for malaria diagnostics [58]. The following section will explore these developments in greater detail.

### Adopting strategies developing radiopharmaceuticals for infection imaging as input for malaria imaging

Historically, radiopharmaceuticals targeting specific microorganism have been pursued to enable precise imaging and diagnoses. For instance, the mechanisms of radiolabelled antibiotics and anti-mycobacterial drugs have been employed successfully for locating the infection site with imaging [59–62]. While radiolabelling existing, approved antibiotics leverages established chemistry and target interactions, it is important to note that pharmacologically active compounds may disrupt normal cellular functions or even eliminate pathogens, potentially interfering with imaging signals [36]. It is well understood that substituting certain elements in drugs — such as carbon (C), nitrogen (N), oxygen (O), and fluorine (F) — with gamma- or positron-emitting isotopes allows the compound to retain its chemical structure and biological activity. This enables in vivo investigations of drug kinetics, dosimetry, and distribution [63]. Although anti-malarial drugs have previously been radiolabelled for preclinical assessments of pharmacokinetics, excretion, and neurotoxicity, no studies have reported their use in pathogen-specific PET- or SPECT imaging [64–67]. Recent advancements in pathogen-specific metabolic radiopharmaceuticals have shown promising results in human trials. For example, Yao et al. (2016) developed an automated Fluorine-18 labelling procedure for 2-deoxy-2-**[**^**18**^**F]fluoro-D-sorbitol**, which effectively targets most *Enterobacteriaceae* during nuclear imaging evaluations [68, 69]. Additionally, 1-deoxy-1-[^18^F]fluoro-D-mannitol has demonstrated preclinical efficacy in distinguishing infection from inflammation, suggesting potential for clinical translation s [70]. These principles could be adapted for imaging *Plasmodium* parasite in vivo. A variety of radiolabelled vitamins and amino acids have been assessed both clinically and preclinically as imaging agents for cancer and other diseases [71–73]. The survival and proliferation of *P. falciparum* parasites depend on host-derived essential nutrients, including isoleucine, methionine, pantothenate (vitamin B5), lipids, and purines [74]. Given that **[**^**11**^**C]methionine** is widely used as an amino acid PET tracer for brain tumour evaluation, it could be explored for malaria imaging [75–78]. Previous in vitro studies have investigated isoleucine and pantothenate uptake in parasite-infected erythrocytes using **[**^**14**^**C]isoleucine** and **[**^**14**^**C]pantothenate** [79, 80]. Additionally, Carbon-11 amino acid labelling of carboxyl groups has been reported and could be adapted for labelling isoleucine, enabling preclinical in vivo imaging assessments. Neuroinflammation arises in response to various stimuli, including brain injury, infection, autoimmune disorders, and peripheral organ inflammation. Microglial cells, the brain’s intrinsic defense mechanism, play a pivotal role in mediating neuroinflammation. Upon activation, translocator protein 18kDa (TSPO; refer to previous notes on macrophage activation in Table 3) is significantly upregulated and expressed on the outer mitochondrial membrane of microglia [81]. TSPO-targeting radiopharmaceuticals have been previously developed, with [^11^C](R)-PK11195 remaining the most widely used [81]. Postmortem analyses of individuals with cerebral malaria (CM) have confirmed microglial activation, and preclinical studies further support its role in CM pathogenesis [82–87]. Consequently, [^11^C](R)-PK11195 and other TSPO-binding radiotracers may be explored for imaging CM-related neuroinflammation, offering a non-invasive tool to assess drug treatment efficacy. Additionally, activated microglia have been reported to induce cyclooxygenase-2 (COX-2) expression, a key enzyme in neuroinflammatory pathways [88]. Overexpression of COX-2 is associated with the pathogenesis of inflammation in the brain and is considered a potential target for in vivo imaging of neuroinflammation. Radiolabelled COX-2 inhibitors, also known as coxibs, such as **[**^**11**^**C]MC1**, **[**^**11**^**C]TMI**, **or [**^**18**^**F]MTP**, have been evaluated preclinically as neuroinflammatory markers [89]. Studies have demonstrated that *P. falciparum* can suppress COX-2-mediated prostaglandin E2 expression, exacerbating disease severity during pregnancy and in children with CM or SMA [90, 91]. COX-2-specific radiolabelled tracers may provide valuable information with regards to neuroinflammation during CM. Sequestration of parasite-infected erythrocytes in organs, including the brain, leads to **reduced blood flow** and **tissue hypoxia**. Among various imaging techniques for detecting hypoxia, research has primarily focused on radiolabelled nitroimidazoles, particularly **[**^**18**^**F]fluoro-misonidazole** (**[18 F]FMISO**), which accumulates in hypoxic tissues. For instance, **[**^**18**^** F]FMISO** (also **[**^**68**^**Ga]Ga-nitroimidazole)** had been successfully utilised for PET-based hypoxia imaging in tuberculosis [92, 93]. This approach can be translated to investigate the development of malaria-associated hypoxia, the pathology of hypoxia, and to monitor adaptive response of the body to treatment. This approach could be adapted to investigate malaria-associated hypoxia, elucidate its pathological mechanisms, and monitor the body’s adaptive response to treatment. By leveraging hypoxia-specific radiopharmaceuticals, researchers may gain deeper insights into malaria progression and therapeutic efficacy.

**Table 3:** Selection of targets involved in malaria pathogenesis for R&D of new radiopharmaceuticals

**Pathway/Interaction**	**Cellular Target**	**Function**	**Possible Imaging Strategy**	Compounds for RP Development
**Malaria Life Cycle**				
Haemoglobin degradation	**Cysteine/aspartic proteases**	Enzymatic breaking down of haemoglobin within iRBCs	Visualise parasitic activity/avidity	Enzyme substrates
Host-pathogen markers	Erythrocyte membrane alterations [46]	iRBCs exhibiting structural changes for needed for survival in the host cell	Visualise cell interaction/-trafficking by direct or indirect cell radiolabelling	Whole red blood cell **[**^**99 m**^**Tc]Tc-iRBC** [47]
Parasite-specific proteins	Plasmodium Heat shock protein 90 (PfHsp90)	Parasite survival/stress response [48]	Imaging parasite proliferation	PfHsp90 (or its binders)
	Circumsporozoite protein 1 (CSP-1)	Mediates sporozoite adhesion to and invasion of human hepatocytes [49]	Imaging parasite – liver interactions	anti–CSP-1 mAb or mAb-L9(LS)
	Plasmodium kinases	Regulate parasite growth and replication	Visualise parasite activity	Enzyme substrates
Drug resistance mechanisms	Transporter proteins	Drug resistance-associated transporters	Imaging MoA through ligand/drug radiolabelling	Drugs/Ligands
	Metabolic pathways	Parasite’s unique metabolic adaptations, such as lipid biosynthesis and nucleotide	Visualise interaction of various *Plasmodium*’s metabolic pathways	Small molecules
**Malaria-Infected Red Blood Cell (iRBC)**				
Surface proteins unique to iRBCs	*P. falciparum* erythrocyte membrane surface protein 1 (PfEMP1)	Key protein involved in cytoadherence/disease severity [50]	Imaging of iRBC distribution/sequestration or organ/tissue pathology	PfEMP1-selective ligands (mAb/mAb variants)
	Ring-infected erythrocyte surface antigen (RESA)	Stabilises the iRBC membrane [51]	Imaging and localisation of disease by iRBC-mediated distribution	RESA-selective ligands (mAb/mAb variants)
	Variant surface anti-gen 2 chondroitin sulfate A (VAR2_CSA_)	Parasite protein variant associated with placental malaria [52]	Specialised malaria imaging	VAR2_CSA_-selective ligands (mAb/mAb variants)
	Targeting sulfonyl-urea receptor 1 by glibenclamide (glib)	To be determined	Imaging/monitoring of replicating parasites within human red blood cells	Glibenclamide [53]
Micro-vesicles and extracellular vesicles	Parasite-derived micro-vesicles	Vesicles carry host and parasite proteins	Imaging early malaria infection (cellular response)	Whole vesicles or vesicle-bound proteins
		Micro-vesicles containing parasite RNA, which activate immune responses	Imaging early malaria infection (host response)	Binders to parasite-derived RNA
Metabolic and transport proteins on iRBC	Glucose transporters	Malaria parasites can alter blood cell glucose uptake	Imaging altered blood cell glucose metabolism	[^18^F]FDG/other sugars with GLUT affinity
	Ion channels/pumps	Malaria parasites alter ion glucose uptake	Imaging altered ion transport in iRBCs	Ion transport substrates
**Malaria-triggered host immune responses**				
Inflammatory cytokines and immune mediators	Interferon-Gamma (IFN-γ)	IFN-γ -mediated activated macrophages and enhancing parasite clearance	Imaging malaria-associated macrophage activity	IFN-γ pathway ligands/binders
	Tumour necrosis factor-alpha (TNF-α)	Elevated TNF-α is prevalent in severe malaria	Imaging changes to TNF-α	TNF-α pathway ligands/binders
	Interleukin-6 (IL-6)/and − 10 (IL-10)	IL-6 promotes the differentiation of naïve CD4^+^ T cells control parasitaemia. IL-10 suppresses inflammation by inhibiting T cell functions.	IL-6 mediated imaging of malaria severity levels. IL-10 adapted Imaging malaria-associated inflammation	IL-6/IL-10
Iron metabolism	Hepcidin regulation	Hepcidin degrades ferroportin, a key iron exporter (reduces iron release into the bloodstream)	Imaging of malaria through induced inflammation	Hepcidin-peptide, Mini-hepcidin analogues
	Plasmodium vivax reticulocyte binding protein 2b (PvRBP2b)	Parasite uses PvRBP2b, an adhesin protein, to bind to TfR1 on reticulocytes, for entering the host cells [54]	Imaging malaria infection through iron metabolism	Transferrin receptor (TfR1)- targeting probes
Cellular immune response (macrophage activation)	Urokinase-type plasminogen activator (uPAR/CD87)	Antigen (polypeptide) is overexpressed upon exposure to inflammatory mediators	Visualisation of macrophages activity	CD87 binders mAb-IIIB6 **[**^**89**^**Zr]Zr-IIIB6** [55]
	Macrophage-bound translocator protein (TSPO)	Target activated macrophages and are responsible for elimination of the parasite	Imaging of MA-ARDS proposed	TSPO binders **[**^**18**^**F]FEPPA** [56]
Other cellular immune response	Dendritic cell function	Cells presenting antigens during malaria	Imaging dendritic cell function (inflammatory processes)	Cell targeting mAb/mAb variants
Vascular or endothelial dysfunction	Intercellular adhesion molecule 1 (ICAM-1)	Involved in parasite cytoadherence/disease severity	ICAM-1-derived visualisation of parasite sequestration processes	ICAM-1 ligands
	Overexpressed vascular cell adhesion molecule (VCAM-1)	Involving endothelial activation during inflammation and vascular dysfunction	Imaging malaria-associated inflammation	VCAM-1 binders
	Blood-brain barrier (BBB) integrity	CM causes BBB disruption	Specialised imaging/monitoring of CM	Small molecules
Altered pulmonary blood flow (capillary dysfunction)	Macroaggregated albumin (MAA)	MAA become temporarily lodged in the pulmonary micro-capillaries	Imaging of malaria-induced pulmonary insufficiency (MA-ARDS)	**[**^**99 m**^**Tc]Tc-MAA** [57]

*iRBCs* Infected red blood cells; *RP* radiopharmaceuticals; *CM* cerebral malaria; *MA-ARDS* malaria-associated acute respiratory distress syndrome; *mAb* monoclonal antibody; *MoA* mechanism of action

### Employing the right animal model for radiopharmaceutical development

Choosing the appropriate animal model is a critical factor in preclinical radiopharmaceutical studies, as it must accurately replicate the infected human system or organ under investigation. Simwela et al. have recently reviewed various animal models used in malaria research, including birds, bats, rodents, and non-human primates (NHPs) [94]. Among these, NHPs infected with *Plasmodium falciparum* isolates are widely used for studying human malaria, offering valuable insights into parasite biology and pathogenesis. NHPs develop malaria with physiological and pathological characteristics similar to those observed in humans, making them highly relevant for translational studies [95]. However, employing NHPs for in vivo imaging presents several challenges, including high costs, complex housing requirements, specialised imaging equipment, the need for multidisciplinary expertise, and stringent ethical considerations. As a more accessible alternative, rodent models are frequently utilised, with certain rodent malaria parasites — such as *P. berghei*, *P. yoelii*, *P. chabaudi*, and *P. vinckei* — demonstrating genetic and physiological similarities to human malaria parasites. These models have been successfully adopted for nuclear imaging applications. While existing malaria animal models are undeniably valuable and widely employed, the effective translation of preclinical findings into clinical malaria diagnostics requires further multidisciplinary research, particularly in advanced imaging techniques. Additionally, a persistent challenge across all preclinical models is the biological variability in parasite stages, which may differ from the presentation of malaria in humans. Addressing these complexities through comparative and integrative studies will enhance the reliability of imaging methodologies for malaria research.

### Clinical assessment of radiotracers: ethical and practical considerations

Clinical trials are essential for developing new tracers, drugs, and vaccines, with final-phase studies involving participants diagnosed with the target disease. However, for infectious diseases, challenge studies —where healthy individuals are deliberately infected — are sometimes necessary to evaluate prevention and treatment strategies. In high-mortality diseases like malaria, such studies must be ethically justified, ensuring maximum societal benefit while prioritising volunteer safety. Due to ethical constraints, malaria research has largely relied on controlled human malaria infection (CHMI) models and preclinical studies [94]. CHMI, where humans are infected by sporozoites injection or by mosquito bites, has been instrumental in developing antimalarial drugs and vaccines [96], While CHMI has been applied to malaria diagnostics and parasite biology studies, its suitability for assessing malaria-specific radiotracers remains debated. A potential solution is integrating radiotracer evaluation into existing malaria-induced studies assessing antimalarial drug efficacy. This approach would allow simultaneous assessment of a tracer’s ability to monitor treatment effects, potentially classifying malaria-specific tracers under diagnostic imaging.

## Future contributions of nuclear imaging to malaria research and diagnostics

Nuclear medicine techniques offer significant potential in malaria research and diagnostics, particularly in advanced detection, disease progression monitoring, and drug efficacy assessment. While not yet suitable during routine screening, PET and SPECT can focus on providing functional and molecular insights into malaria pathophysiology, aiding in the development of targeted interventions. Cerebral malaria, a severe complication of *P. falciparum* infection, disrupts the blood-brain barrier, leading to neurological damage. PET/SPECT imaging can be employed to investigate these disruptions, with radiolabelled tracers targeting endothelial activation markers such as **ICAM-1** and **VCAM-1** [97]; future imaging techniques could improve early detection and therapeutic monitoring of CM. As **iRBCs** have demonstrated altered haemoglobin degradation and nutrient uptake, strategically placed nuclear imaging can help analyse these changes, with potential radiopharmaceuticals targeting glucose transporters or ion channels providing insights into parasite survival mechanisms. Studies have demonstrated the feasibility of metabolic imaging in infectious diseases, which could be adapted for malaria [98].

Most SM patients clinically show microvascular sequestration, causing complications in organs like the liver and lungs. Nuclear imaging strategies could be employed in the future to map parasite sequestration sites, assess the severity of malaria pathophysiology, and refine treatment approaches. Studies on vascular dysfunction imaging in infectious diseases have been reviewed and may help translate PET/SPECT imaging into a valuable tool for understanding malaria-associated microvascular damage [99, 100]. Furthermore, PET imaging has been successfully applied in assessing drug resistance in bacterial infections, suggesting its potential applicability in malaria research. Reports on antimalarial drug efficacy, resistance, and response emphasise the need for continuous monitoring to adapt treatment protocols and improve patient outcomes [101]. Radiolabelled inhibitors may help detect metabolic shifts and drug resistance markers, aiding in the development of more effective therapies. PET imaging has been successfully used to assess drug resistance in bacterial infections, suggesting its applicability in malaria research [102].

## Conclusive statements

This review highlights the current role of nuclear imaging and emphasises on advancing nuclear imaging research in malaria diagnostics to inspire further investigations in this field. Studies indicate that MRI and US are widely used for assessing deep-seated malaria infections, but they primarily rely on detecting structural abnormalities caused by the host response or tissue damage. Differentiating between various diseases, inflammation, and oncologic processes remains a challenge with these techniques. Although nuclear medicine is not yet practical for routine malaria screening, its specialised imaging capabilities could play a crucial role in severe or drug-resistant malaria cases. PET and SPECT imaging can provide molecular-level insights into parasite sequestration, cerebral malaria pathology, and vascular dysfunction, enabling early detection and treatment monitoring. Despite progress in nuclear imaging techniques, there remains a critical need for parasite-specific radiotracers and integrating malaria-specific imaging into the existing diagnostic frameworks could refine anti-malarial drug development, offering a more comprehensive approach to disease management. Such development has proven valuable in other infectious diseases, suggesting that similar approaches could be applied to malaria. Encouraging researchers to design radiotracers targeting *Plasmodium* or malaria-specific tissue responses could complement host-response-dependent detection methods, expand diagnostic capabilities, and improve SM outcome predictions.

## Electronic supplementary material

Below is the link to the electronic supplementary material.

Supplementary Material 1

Supplementary Material 2
